# Mice Lacking γδ T Cells Exhibit Impaired Clearance of Pseudomonas aeruginosa Lung Infection and Excessive Production of Inflammatory Cytokines

**DOI:** 10.1128/IAI.00171-20

**Published:** 2020-05-20

**Authors:** Toka Omar, Pascal Ziltener, Erin Chamberlain, Zhenyu Cheng, Brent Johnston

**Affiliations:** a Department of Microbiology and Immunology, Dalhousie University, Halifax, Nova Scotia, Canada; b Department of Pediatrics, Dalhousie University, Halifax, Nova Scotia, Canada; c Department of Pathology, Dalhousie University, Halifax, Nova Scotia, Canada; Georgia Institute of Technology School of Biological Sciences

**Keywords:** *Pseudomonas aeruginosa*, gamma delta T cell

## Abstract

Pseudomonas aeruginosa is an opportunistic pathogen that causes chronic and life-threatening infections in immunocompromised patients. A better understanding of the role that innate immunity plays in the control of P. aeruginosa infection is crucial for therapeutic development. Specifically, the role of unconventional immune cells like γδ T cells in the clearance of P. aeruginosa lung infection is not yet well characterized. In this study, the role of γδ T cells was examined in an acute mouse model of P. aeruginosa lung infection. In the absence of γδ T cells, mice displayed impaired bacterial clearance and decreased survival, outcomes which were associated with delayed neutrophil recruitment and impaired recruitment of other immune cells (macrophages, T cells, natural killer cells, and natural killer T [NKT] cells) into the airways. Despite reduced NKT cell recruitment in the airways of mice lacking γδ T cells, NKT cell-deficient mice exhibited wild-type level control of P. aeruginosa infection. Proinflammatory cytokines were also altered in γδ T cell-deficient mice, with increased production of interleukin-1β, interleukin-6, and tumor necrosis factor. γδ T cells did not appear to contribute significantly to the production of interleukin-17A or the chemokines CXCL1 and CXCL2. Importantly, host survival could be improved by inhibiting tumor necrosis factor signaling with the soluble receptor construct etanercept in γδ cell-deficient mice. These findings demonstrate that γδ T cells play a protective role in coordinating the host response to P. aeruginosa lung infection, both in contributing to early immune cell recruitment and by limiting inflammation.

## Abstract (precis)

Pseudomonas aeruginosa is an opportunistic pathogen that causes chronic and life-threatening infections in immunocompromised patients. A better understanding of the role that innate immunity plays in the control of P. aeruginosa infection is crucial for therapeutic development. Specifically, the role of unconventional immune cells like γδ T cells in the clearance of P. aeruginosa lung infection is not yet well characterized.

## INTRODUCTION

Pseudomonas aeruginosa is a Gram-negative, rod-shaped bacterium found ubiquitously in the environment. It is an opportunistic pathogen that commonly infects immunocompromised individuals, especially in hospital settings (2). It is also the leading cause of morbidity and mortality in cystic fibrosis (3). By late adolescence, 80% of cystic fibrosis patients are chronically infected with P. aeruginosa (4). In recent years, the rapid emergence of multidrug-resistant P. aeruginosa necessitates an urgent need for new treatments for the infections caused by this bacterial pathogen. One potential strategy to control P. aeruginosa infections would be to boost protective aspects of host immunity. A better understanding of the cellular mechanisms involved in host defense against P. aeruginosa infection will facilitate the development of such therapies.

The innate immune response plays an important role in the host defense against P. aeruginosa infection. An important aspect of the host defense response is the secretion of proinflammatory cytokines like tumor necrosis factor (TNF), interleukin-6 (IL-6), and IL-1 that facilitate immune cell recruitment to the site of infection. For example, TNF is a strong mediator of inflammatory and immune functions and is produced by monocytes, macrophages, T cells, natural killer (NK) cells, and neutrophils upon bacterial infection (5). Lee et al. reported that TNF knockout mice failed to recruit neutrophils to the airways after P. aeruginosa infection (6).

Rapid and robust recruitment of neutrophils is a hallmark of P. aeruginosa lung infection and is crucial for bacterial pathogen clearance. In a mouse model of P. aeruginosa lung infection, neutrophil depletion rendered mice susceptible to a very low inoculum of several different P. aeruginosa strains (7). The primary role of recruited neutrophils is pathogen elimination through neutrophil serine proteases like neutrophil elastase (8, 9) and generation of reactive oxygen and nitrogen species (10). Other immune cells are also involved in the resolution of P. aeruginosa lung infection. For example, alveolar macrophages are not only responsible for the internalization and killing of the bacterial pathogen but also the phagocytosis of dying neutrophils, thus limiting neutrophil-induced tissue damage (11). NK cells and NKT cells are innate immune cells that recognize stress proteins induced on infected cells via NKG2D receptors and help clear pathogens via production of interferon gamma (IFN-γ) (12).

γδ T cells play an important role in regulating the initial immune response to lung infections caused by various bacterial pathogens, such as Mycobacterium tuberculosis (13), Streptococcus pneumoniae (14), or Staphylococcus aureus (15). Following S. aureus infection, accumulation of γδ T cells in the lungs was reported to mediate bacterial clearance and neutrophil recruitment through the production of IL-17 (15). However, the role of γδ T cells in proinflammatory cytokine production and immune cell recruitment against P. aeruginosa lung infection is not well characterized.

The objective of the present study was to elucidate the role of γδ T cells in defense of the lung against P. aeruginosa challenge *in vivo*. To study the contribution of γδ T cells, various immune parameters were measured in wild-type and γδ T cell-deficient TCRδ^−/−^ mice following P. aeruginosa lung infection. TCRδ^−/−^ mice exhibited decreased bacterial clearance and survival, increased proinflammatory cytokine production, as well as delayed neutrophil infiltration upon intranasal challenge with P. aeruginosa strain K (PAK). Survival could be extended by inhibiting TNF signaling with the soluble receptor construct etanercept. These data implicate an important role for γδ T cells in regulating the host response to P. aeruginosa lung infection.

## RESULTS

### Reduced survival in TCRδ^−/−^ mice upon intranasal challenge with P. aeruginosa.

**FIG 1:**
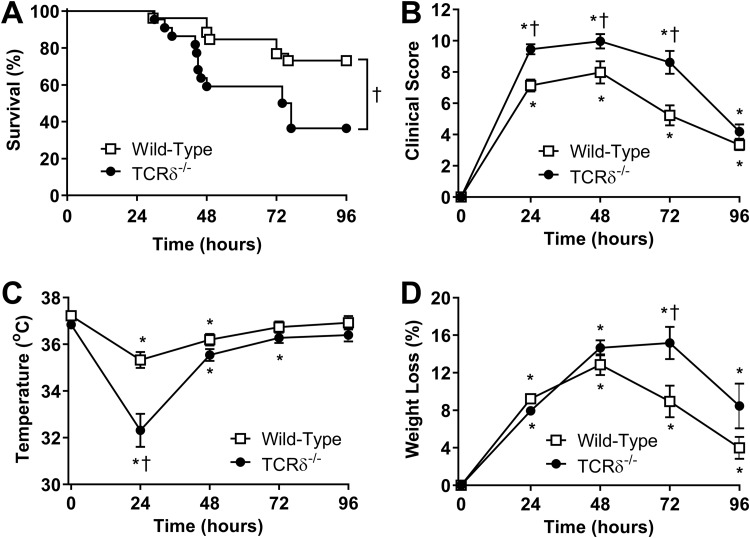
Survival and clinical parameters in wild-type C57BL/6 and TCRδ^−/−^ mice infected with P. aeruginosa. Survival curves (A), clinical scores (B), rectal temperature (C), and weight loss (D) were measured in wild-type and TCRδ^−/−^ mice intranasally inoculated with 1.8 × 10^7^ CFU PAK (*n* = 22 to 26 per group, pooled from 4 separate experiments). Survival curves were compared by Mantel-Cox log-rank test. Other parameters were assessed by Tukey’s multiple-comparison test. *, *P* < 0.05 compared with time zero; †, *P* < 0.05 compared with wild-type mice.

To test the biological impact of γδ T cells in host defense against P. aeruginosa infection, wild-type and TCRδ^−/−^ C57BL/6 mice were infected intranasally with 1.8 × 10^7^ CFU of PAK. Clinical scores and survival were assessed over the course of 4 days. The survival rate at 96 h post-PAK infection was approximately 73% in wild-type mice but only 36% in TCRδ^−/−^ mice (Fig. 1A). This was coupled with a greater increase in overall clinical scores (Fig. 1B), decreased core body temperature (Fig. 1C), and increased weight loss (Fig. 1D) in TCRδ^−/−^ mice. These data reveal an important role for γδ T cells in host defense against P. aeruginosa lung infection.

### Increased bacterial load in lungs of TCRδ^−/−^ mice following P. aeruginosa lung infection.

**FIG 2:**
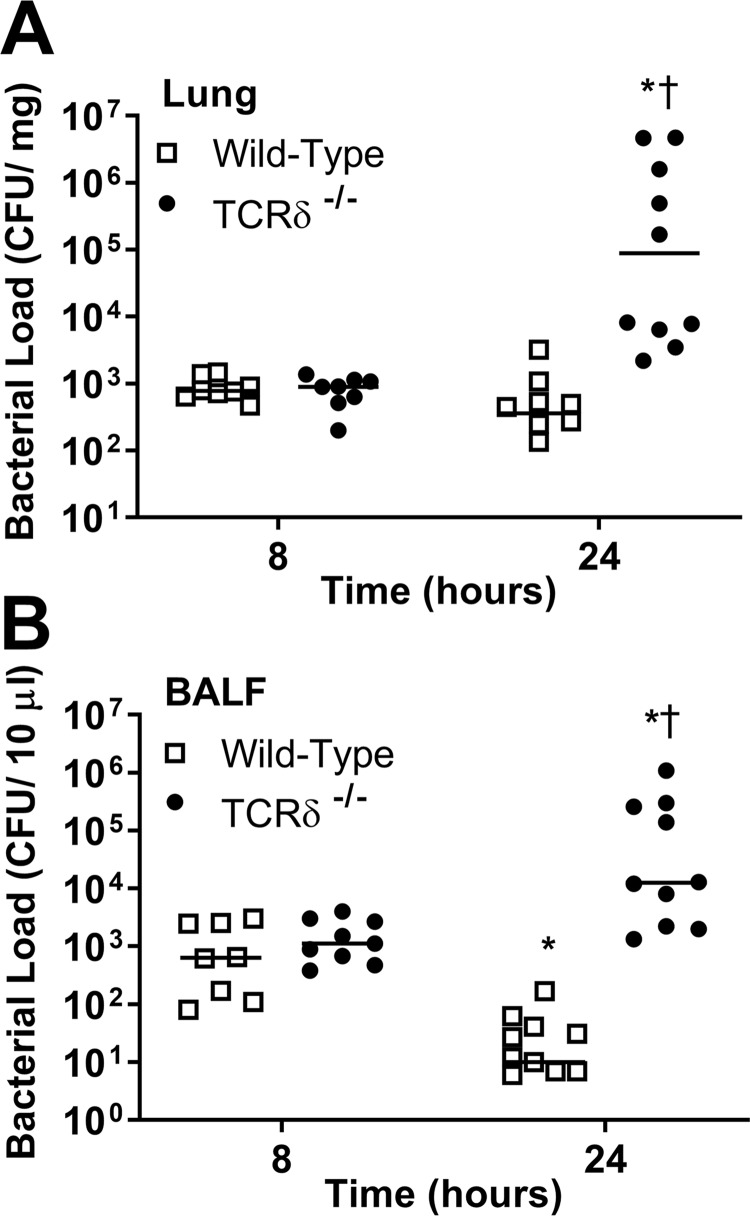
Bacterial load in wild-type C57BL/6 and TCRδ^−/−^ mice infected with P. aeruginosa. Wild-type and TCRδ^−/−^ mice were infected intranasally with 1.8 × 10^7^ CFU PAK. CFU were evaluated in lung homogenates (A) and BALF (B) at 8 or 24 h after infection (*n* = 8 to 10 per group). Each symbol represents an individual animal, and horizontal lines represent the median. *, *P* < 0.05 compared with 0 h; †, *P* < 0.05 compared with wild-type mice (using Dunn’s multiple-comparison test).

To determine the influence of γδ T cells on clearance of PAK from the lungs, the bacterial load was examined in lung tissue and bronchoalveolar lavage fluid (BALF) of wild-type and TCRδ^−/−^ mice at 8 h and 24 h postinfection. The bacterial CFU in the lungs and BALF of wild-type and TCRδ^−/−^ mice were similar 8 h after infection. However, the bacterial burden in the lungs and BALF of TCRδ^−/−^ mice was significantly greater at 24 h postinfection (Fig. 2A and B). In contrast, the bacterial burden in wild-type mice remained unchanged in the lung tissue and decreased significantly in the BALF at 24 h (Fig. 2A and B). Interestingly, the bacterial load was much higher in a subset of TCRδ^−/−^ mice. All mice that succumbed to PAK infection (wild-type and TCRδ^−/−^) exhibited increased bacterial load at necropsy (data not shown). However, since bacterial load determination is an endpoint assay, we could not test directly whether enhanced bacterial load correlates with reduced survival. These results indicate that γδ T cells play an important role in regulating bacterial clearance, which may improve survival during P. aeruginosa lung infection.

### Altered immune cell recruitment in TCRδ^−/−^ mice following P. aeruginosa lung infection.

**FIG 3:**
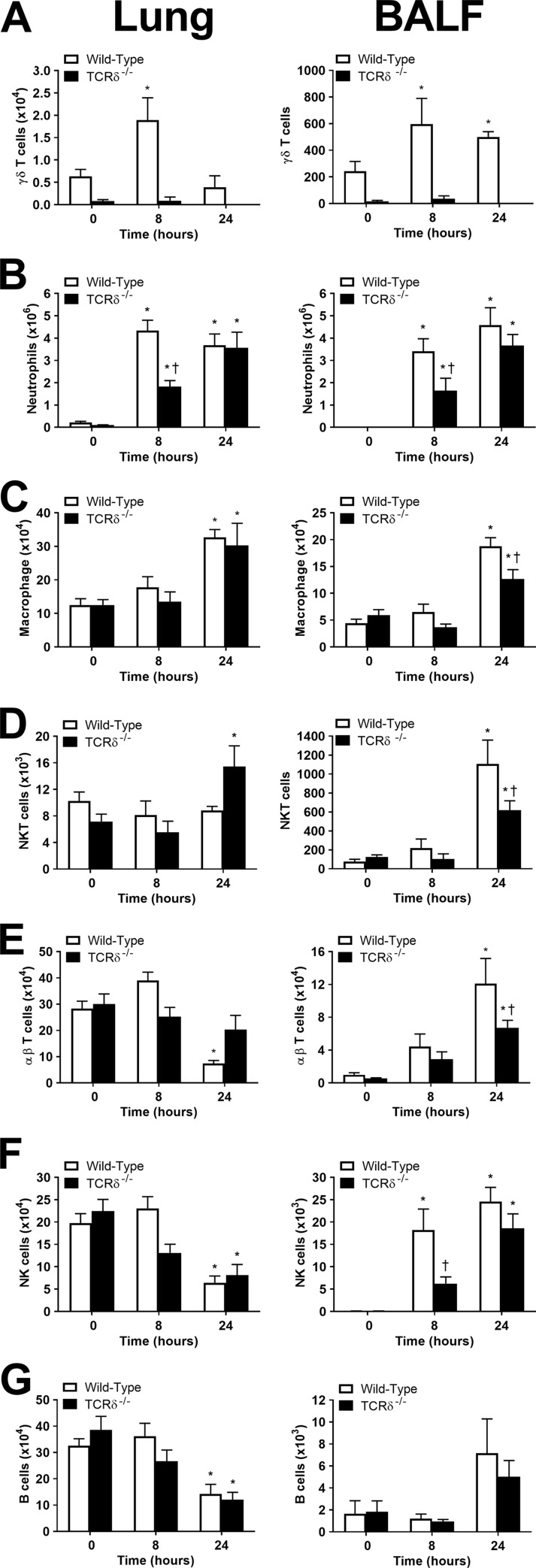
Immune cell recruitment in wild-type C57BL/6 and TCRδ^−/−^ mice infected with P. aeruginosa. Wild-type and TCRδ^−/−^ mice were infected intranasally with 1.8 × 10^7^ CFU PAK. The numbers of γδ T cells (A), neutrophils (B), macrophages (C), NKT cells (D), αβ T cells (E), NK cells (F), and B cells (G) were measured by flow cytometric analysis of lung and BALF cells using specific surface markers for each cell type (*n* = 8 to 12 per group, pooled from 3 separate experiments). *, *P* < 0.05 compared with 0 h; †, *P* < 0.05 compared with wild-type mice (using Tukey’s multiple-comparison test).

To evaluate the role of γδ T cells in regulating immune cell recruitment, we compared the number and types of immune cells present at 0 h (uninfected), 8 h, and 24 h after PAK infection. γδ T cells were detected in the lungs and BALF of uninfected wild-type C57BL/6 mice, and the number of γδ T cells increased at 8 h following infection with PAK (Fig. 3A). γδ T cell numbers in the lung returned to baseline at 24 h while remaining elevated in the BALF, suggesting movement into the airways. In contrast, only low levels of background antibody staining were detected in the lungs and BALF of TCRδ^−/−^ mice (Fig. 3A), validating the lack of γδ T cells in TCRδ^−/−^ mice.

One of the essential factors contributing to P. aeruginosa clearance is the recruitment of neutrophils and other immune cells to the site of infection (7). Compared to wild-type mice, significantly fewer neutrophils infiltrated the lungs and BALF of TCRδ^−/−^ mice at 8 h postinfection; however, neutrophil infiltration in wild-type and TCRδ^−/−^ mice was not different at 24 h postinfection (Fig. 3B), suggesting a delay in neutrophil recruitment in the absence of γδ T cells. There was increased recruitment of macrophage-like cells into the lungs of wild-type and TCRδ^−/−^ mice at 24 h postinfection, but TCRδ^−/−^ mice exhibited significantly reduced macrophage recruitment into the BALF at 24 h postinfection (Fig. 3C).

There was no change in the number of NKT cells in the lungs of wild-type mice, but a significant increase was observed in the lungs of TCRδ^−/−^ mice at 24 h (Fig. 3D). In the BALF, NKT cells were increased at 24 h in both wild-type and TCRδ^−/−^ airways but significantly more so in wild-type mice (Fig. 3D), suggesting an impairment in movement of NKT cells from the lung into the airways. PAK infection of NKT cell-deficient Jα18^−/−^ mice did not result in increased mortality (see Fig. S1 in the supplemental material) or impaired bacterial control (see Fig. S2 in the supplemental material), indicating that NKT cells are not required for the control of PAK.

αβ T cells were decreased in the lungs and increased in the BALF of wild-type mice at 24 h (Fig. 3E). In TCRδ^−/−^ mice, αβ T cells did not decrease significantly in the lung and did not increase in the BALF to the extent observed in wild-type mice. The number of NK cells in the lungs was decreased at 24 h after infection and increased in the BALF of wild-type mice by 8 h (Fig. 3F). Accumulation of NK cells in the BALF was delayed in TCRδ^−/−^ mice. B cells were decreased in the infected lungs of both wild-type and TCRδ^−/−^ mice by 24 h (Fig. 3G). The number of B cells in the BALF tended to increase at 24 h but did not reach statistical significance (Fig. 3G). Overall, the loss of γδ T cells resulted in delayed recruitment of neutrophils to the lung and impaired immune cell infiltration into the airways.

### Neutrophil-recruiting chemokines are not altered in TCRδ^−/−^ mice following P. aeruginosa lung infection.

**FIG 4:**
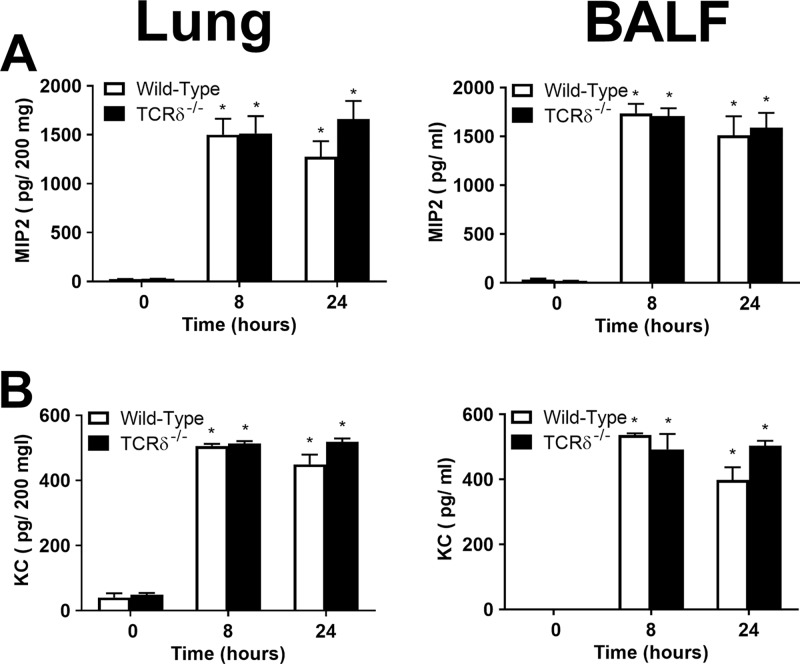
Chemokine production in wild-type C57BL/6 and TCRδ^−/−^ mice infected with P. aeruginosa. Wild-type and TCRδ^−/−^ mice were infected intranasally with 1.8 × 10^7^ CFU PAK. The chemokines CXCL2 (MIP2) (A) and CXCL1 (KC) (B) were measured in lung homogenates and BALF at 0 (untreated), 8, or 24 h postinfection with P. aeruginosa (*n* = 6 per group at 0 h, 9 or 10 per group at 8 h, and 6 or 7 per group at 24 h, pooled from 3 separate experiments). *, *P* < 0.05 compared with 0 h; †, *P* < 0.05 compared with wild-type mice (using Tukey’s multiple-comparison test).

As neutrophil recruitment was reduced at early time points following P. aeruginosa lung infection, we examined the levels of CXCL1 (KC) and CXCL2 (MIP-2), chemokines that have been implicated in neutrophil recruitment during P. aeruginosa infection (16). Levels of these chemokines were increased equally in both wild-type and TCRδ^−/−^ mice 8 and 24 h after PAK infection (Fig. 4A and B). We cannot exclude the possibility that these chemokines or other chemoattractants were altered in TCRδ^−/−^ mice at earlier time points.

### Increased proinflammatory cytokine production in TCRδ^−/−^ mice following P. aeruginosa lung infection.

**FIG 5:**
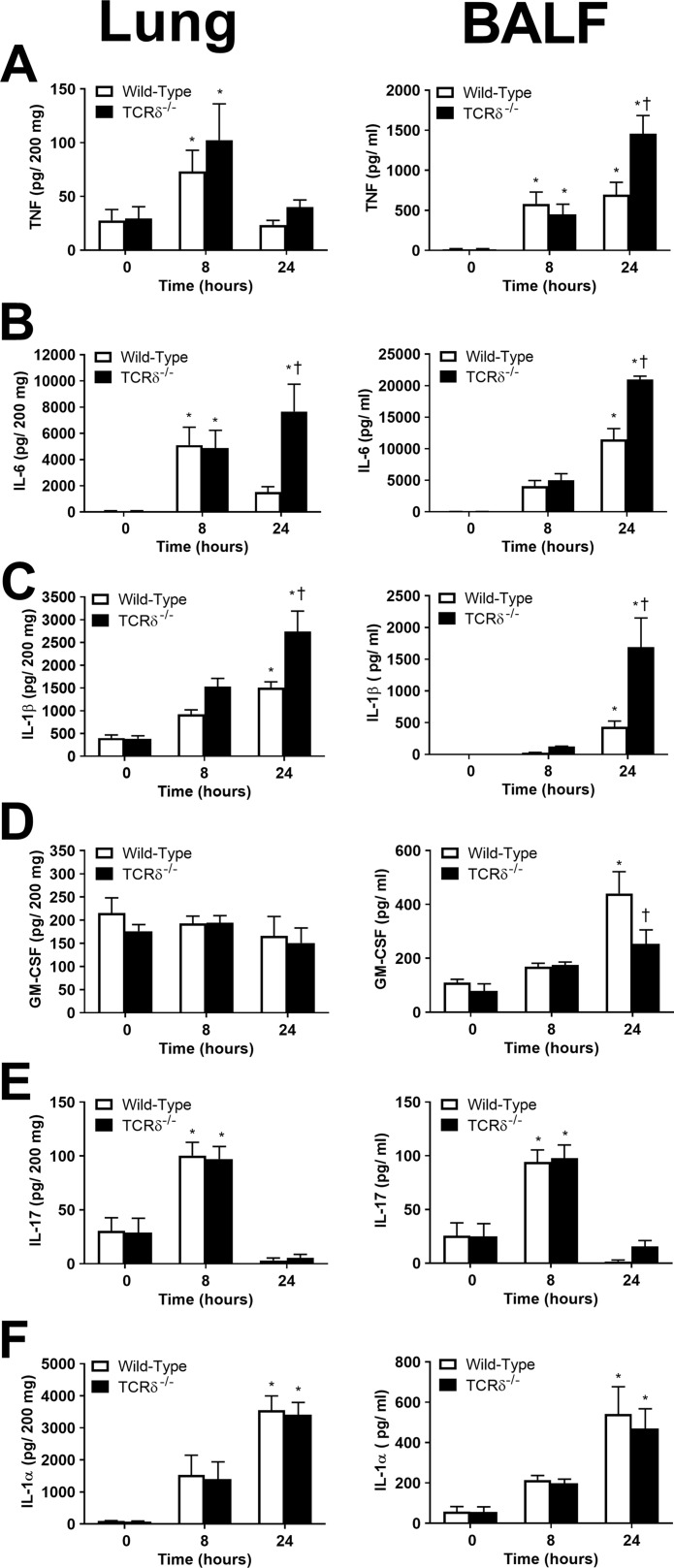
Cytokine production in wild-type C57BL/6 and TCRδ^−/−^ mice infected with P. aeruginosa. Wild-type and TCRδ^−/−^ mice were infected intranasally with 1.8 × 10^7^ CFU PAK. The cytokines TNF (A), IL-6 (B), IL-1β (C), GM-CSF (D), IL-17 (E), and IL-1α (F) were measured in lung homogenates and BALF at 0 (untreated), 8, or 24 h postinfection with P. aeruginosa (*n* = 6 or 7 per group at 0 h, 9 or 10 per group at 8 h, and 11 or 12 per group at 24 h, pooled from 3 separate experiments). *, *P* < 0.05 compared with 0 h; †, *P* < 0.05 compared with wild-type mice (using Tukey’s multiple-comparison test).

Local production of cytokines in the lungs influences host defense mechanisms against P. aeruginosa infection (17–19). However, the excessive production of proinflammatory cytokines can lead to tissue damage and other detrimental effects for the host. The levels of secreted cytokines in the lung tissue and BALF of wild-type and TCRδ^−/−^ mice were measured at 0 h (uninfected), 8 h, and 24 h after infection. Consistent with a previous report (20), TNF levels were increased primarily in the BALF compared to the lung (Fig. 5A). Notably, TNF levels in the BALF at 24 h were significantly higher in TCRδ^−/−^ mice than in wild-type mice (Fig. 5A). IL-6 levels were increased in the lungs of both wild-type and TCRδ^−/−^ mice at 8 h postinfection (Fig. 5B). While IL-6 decreased in the lungs of wild-type mice at 24 h, it remained high in TCRδ^−/−^ mice (Fig. 5B). IL-6 levels in the BALF were also increased at 24 h in TCRδ^−/−^ mice compared to those in wild-type mice (Fig. 5B). The levels of the proinflammatory cytokine IL-1β were also significantly higher in the lungs and BALF of TCRδ^−/−^ mice than in wild-type mice (Fig. 5C). Granulocyte-macrophage colony-stimulating factor (GM-CSF), which is required for host survival in P. aeruginosa infection (21), was lower in the BALF of TCRδ^−/−^ mice than in wild-type animals (Fig. 5D).

Consistent with previous studies (22), IL-17A levels in the lung tissue and BALF were increased 8 h after infection and returned to the baseline by 24 h (Fig. 5E). Surprisingly, the levels of IL-17A did not differ between wild-type and TCRδ^−/−^ mice, even though γδ T cells have been reported as a source of IL-17 (23). IL-1α levels in the lung and BALF increased over time but were not significantly different between wild-type and TCRδ^−/−^ mice (Fig. 5F). Similarly, the levels of IL-2, IL-4, IL-5, IL-10, IFN-γ, and keratinocyte growth factor did not differ between wild-type and TCRδ^−/−^ mice (data not shown). These results demonstrate that the production of some proinflammatory cytokines is altered in the absence of γδ T cells, likely contributing to increased pathogenesis and decreased survival following P. aeruginosa infection.

### Improved survival of PAK-infected TCRδ^−/−^ mice with TNF signaling blockade.

**FIG 6:**
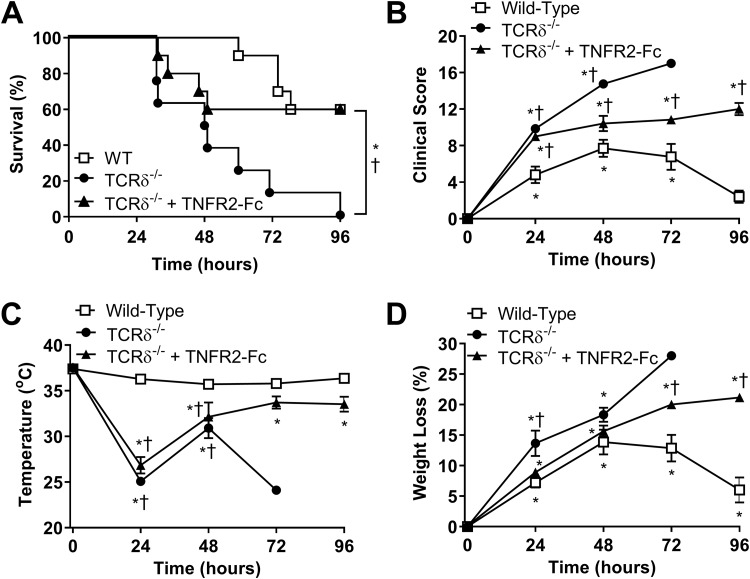
Survival and clinical parameters in P. aeruginosa-infected TCRδ^−/−^ mice treated with etanercept. Survival curves (A), clinical scores (B), rectal temperature (C), and weight loss (D) were measured in wild-type C57BL/6 mice, TCRδ^−/−^ mice, and TCRδ^−/−^ mice treated with etanercept (TNFR2-Fc; 100 μg) following intranasal inoculation with 1.8 × 10^7^ CFU PAK (*n* = 8 to 10 per group, pooled from 2 separate experiments). Survival curves were compared by Mantel-Cox log-rank test. *, *P* < 0.05 compared to WT mice; †, *P* < 0.05 compared to TCRδ^−/−^ mice treated with etanercept. Other parameters were assessed by Tukey’s multiple-comparison test. *, *P* < 0.05 compared with time zero; †, *P* < 0.05 compared with wild-type mice.

As TNF is known to be an early mediator in the inflammatory cytokine cascade (6), we sought to determine whether the excessive TNF production in the BALF of TCRδ^−/−^ mice was detrimental to survival following P. aeruginosa infection. Etanercept, a soluble TNFR2-Fc fusion protein that inhibits mouse and human TNF (24), was administered intraperitoneally 1 h postinfection to block TNF signaling. Overall, blockade of TNF signaling boosted survival in TCRδ^−/−^ mice to the levels observed in infected wild-type mice (Fig. 6A). However, TNF blockade did not prevent the early mortality observed in TCRδ^−/−^ mice. Clinical scores in etanercept-treated TCRδ^−/−^ mice were lower than those of untreated TCRδ^−/−^ mice but remained higher than those of wild-type mice throughout the experimental time course (Fig. 6B). TNF blockade did not prevent the initial decrease in temperature observed in infected TCRδ^−/−^ mice (Fig. 6C). Variability in temperature in untreated mice over time reflects the progressive loss of mice in the experiment; the last mouse in the TCRδ^−/−^ group exhibited a relapse at 72 h and subsequently succumbed to infection. Consistent with the clinical score, TNF blockade resulted in weight loss that was intermediate between wild-type and TCRδ^−/−^ mice (Fig. 6D). Although etanercept prolonged survival in TCRδ^−/−^ mice, these mice did not recover from infection and likely would have succumbed in a longer experimental protocol. It is clear that other factors must also contribute to the increased pathology and mortality observed in PAK-infected TCRδ^−/−^ mice.

## DISCUSSION

γδ T cells are a subset of unconventional T lymphocytes that play important roles in protection against bacterial, viral, and parasitic infections (13–15, 25, 26). In this study, we examined the impact of γδ T cells on innate immune responses during P. aeruginosa pulmonary infection. In the absence of γδ T cells, bacterial clearance was impaired, and survival was significantly decreased. This was associated with delayed neutrophil recruitment and increased proinflammatory cytokine production. These findings demonstrate that γδ T cells play a protective role in coordinating host responses against P. aeruginosa infection.

Early neutrophil recruitment is essential for protection against bacterial infection, resulting in clearance via phagocytosis, protease release, and production of reactive oxygen and nitrogen species (8–10, 27, 28). In neutropenic mice, intranasal P. aeruginosa infection with a dose as low as 10 to 100 CFU is fatal (8). The delayed neutrophil recruitment observed in TCRδ^−/−^ mice likely impairs the innate immune response against P. aeruginosa lung infection, leading to decreased bacterial clearance and reduced survival.

The cytokine IL-17 has been shown to mediate neutrophil recruitment to sites of infection via induction of the chemokines CXCL1 and CXCL2 (18, 29). γδ T cells are known to produce IL-17 (30, 31), but the role of IL-17 producing γδ T cells during pulmonary P. aeruginosa infection is not clear in the literature. Liu et al. (31) showed that IL-17 production was reduced and bacterial load was increased in P. aeruginosa-infected mice depleted of γδ T cells. However, CD4 T cells, B cells, and group 3 innate lymphoid cells also produce IL-17 during P. aeruginosa infection, and γδ T cells were not the major population of IL-17^+^ cells in infected mice (31, 32). In our study, IL-17 production was not disrupted in TCRδ^−/−^ mice (Fig. 5E), confirming that γδ T cells are not the major source of IL-17 during P. aeruginosa infection.

While the neutrophil-recruiting chemokines CXCL1 and CXCL2 can be upregulated by IL-17, they are also upregulated via IL-17-independent mechanisms (20, 33). We measured CXCL1 and CXCL2 following P. aeruginosa lung infection and found no decreases in TCRδ^−/−^ mice that would explain the delayed neutrophil recruitment. However, we cannot exclude the possibility of differences at earlier time points or impaired production of other neutrophil chemoattractants in TCRδ^−/−^ mice.

We made the novel finding that recruitment of other immune cells implicated in defense against P. aeruginosa (macrophages, NKT, NK, and T cells) (34, 35) was also reduced in infected TCRδ^−/−^ mice. This appeared to be due to reduced recruitment of immune cells from the lung tissue into the airways. It is unclear whether this was due to the absence of γδ T cell-derived signals or secondary to the delay in neutrophil recruitment. In support of the latter, NKT cell recruitment out of the lung vasculature during streptococcal infection is dependent on neutrophil-derived signals (36).

As mice deficient in NKT cells were reported to have impaired clearance of P. aeruginosa strain D4 (37), and we observed altered NKT cell recruitment in TCRδ^−/−^ mice, we examined the role of NKT cells in infection with P. aeruginosa PAK. In contrast to the published results with the D4 strain, we did not observe a difference in survival or bacterial load in NKT cell-deficient Jα18^−/−^ mice infected with PAK (see Fig. S1 and S2 in the supplemental material). Our results are consistent with a report showing that NKT cells played little role in the control of P. aeruginosa strain PAO1 (38). It is possible that different P. aeruginosa strains elicit distinct host responses and pathogenesis.

The reduced production of GM-CSF in the BALF of TCRδ^−/−^ mice could also contribute to the impaired clearance of P. aeruginosa and increased mortality observed in these mice. Mechanistically, GM-CSF in the lung enhances the phagocytosis and bacterial killing activities of alveolar macrophage, and GM-CSF-deficient mice succumb to respiratory P. aeruginosa infection (21).

The current data show that proinflammatory cytokines, specifically IL-1β, IL-6, and TNF, are upregulated in the absence of γδ T cells (Fig. 5). Proinflammatory cytokines play a role in bacterial clearance through the amplification of the inflammatory response (39). However, overproduction of these cytokines has detrimental effects on the host, including systemic inflammation and severe tissue damage (40, 41). In this study, etanercept increased survival of TCRδ^−/−^ mice infected by P. aeruginosa, suggesting that the overproduction of TNF in the absence of γδ T cells contributes to increased mortality. While TNF has proinflammatory effects that assist in bacterial clearance (6), the role of TNF in P. aeruginosa clearance is unclear. TNFR1- and TNFR1/TNFR2-deficient mice cleared P. aeruginosa PAK faster than their wild-type counterparts (42), while TNF^−/−^ mice exhibited higher mortality (7). These differences could relate to the disparate genetic backgrounds of the mice used in these studies or uncharacterized receptors for TNF. Different mouse strains exhibit distinct susceptibilities to P. aeruginosa infection (43); therefore, it is important to consider the roles of immune effectors in the context of specific host-pathogen backgrounds.

In summary, our study has shown that γδ T cells play an important role in regulating innate host responses against P. aeruginosa pulmonary infection. γδ T cells facilitated immune cell recruitment and regulated cytokine production during P. aeruginosa challenge, contributing to bacterial clearance and survival. Further characterization of the mechanisms underlying their protective roles during infection will facilitate approaches to modify the host immune response to target hard-to-treat bacterial infections like P. aeruginosa.

## MATERIALS AND METHODS

### Mice.

C57BL/6 mice and γδ T cell-deficient TCRδ^−/−^ mice (44) were purchased from the Jackson Laboratory (Bay Harbor, ME). NKT cell-deficient Jα18^−/−^ mice were generated in the laboratory of M. Taniguchi (RIKEN Research Center for Allergy and Immunology, Kanagawa, Japan) (45). Mice were maintained under specific-pathogen-free conditions in the Carleton Animal Care Facility (Dalhousie University) with *ad libitum* access to food and water. Male wild-type and TCRδ^−/−^ mice were used in experiments at 8 to 12 weeks of age. All animal protocols were approved by the University Committee on Laboratory Animals in accordance with the guidelines of the Canadian Council on Animal Care.

### Preparation of P. aeruginosa and infection.

P. aeruginosa strain K (PAK) was obtained from T. J. Lin (Dalhousie University). A single colony was used to inoculate 5 to 10 ml of LB broth, and the bacterial suspension was grown overnight with shaking at 37°C. Bacteria were resuspended in room temperature phosphate-buffered saline (Sigma-Aldrich) for determination of the optical density at 600 nm (OD_600_), where 1 unit of OD_600_ represents 8 × 10^8^ CFU of PAK culture. Bacteria were resuspended in saline to infect mice with a dose of 1.8 × 10^7^ CFU in 20 μl. Mice were anesthetized intraperitoneally with 60 μl anesthetic (80 mg of ketamine/kg of body weight and 16 mg/kg xylazine) and infected intranasally by placing saline droplets containing PAK onto the nostrils.

### Monitoring mice for survival.

Mice were monitored up to 96 h after infection. Clinical scores were ranked from 0 to 18 based on the parameters shown in Table 1. Rectal temperature was measured using a thermistor probe (YSI 451; Advanced Industrial Systems, Inc.). Hydration was measured by pinching the skin of the mouse between two fingers and observing its return to its original position. Mice were euthanized if weight loss exceeded 20%, balance or mobility was compromised, or total clinical score exceeded 15. In some groups, mice were treated intraperitoneally with the TNFR2-Fc fusion protein etanercept (100 μg per mouse; Enbrel; Immunex Corporation) or an equal volume of saline at 1 h postinfection, followed by monitoring over 96 h.

**TABLE 1:** Clinical scoring criteria for P. aeruginosa infection

Score	Temperature (°C)	Weight loss (from preinfection weight) (%)	Dehydration	Behavior	Posture	Appearance
0	36–37	<5	Normal	Normal	Normal	Normal
1	35–35.9	<10	Mild (<1 s skin tent)	Slightly reduced	Hunched posture	Piloerection
2	34–34.9	<15	Moderate (1–2 s skin tent)	Slow moving, increased effort	Very hunched posture, head resting on floor	Rough coat
3	<34	<20	Severe (>2 s skin tent)	Moves when prodded	Lying prone/unable to maintain upright posture	Rough coat, lack of grooming

### Isolation of lung cells for flow cytometry.

Mice were euthanized at 0 (uninfected), 8, and 24 h postinfection to obtain lungs and BALF. Airways were lavaged 3 times with 1 ml phosphate-buffered saline. Erythrocytes were lysed using lysis buffer (155 mM NH_4_Cl buffer and 10 mM KHCO_3_, pH 7.4) for 5 min. Lung tissue was minced and passed through a 200-gauge stainless steel mesh into Hanks’ balanced salt solution (Invitrogen) containing 5% fetal bovine serum (FBS) (Invitrogen). Lung cells were centrifuged at 863 × *g* through an isotonic 33% Percoll gradient (GE Healthcare), containing 5% FBS and 100 U/ml heparin (Sigma-Aldrich), for 20 min at 20°C. The resulting pellet was incubated in erythrocyte lysis buffer for 5 min. Cells were resuspended in Hanks’ balanced salt solution containing 5% FBS. Cell samples were stained with TCRδ fluorescein isothiocyanate (FITC) (GL3; BD Biosciences), TCRβ phycoerythrin (PE) (H57-597; eBioscience), NK1.1 peridinin chlorophyll protein (PerCP) Cy5.5 (PK136; eBioscience), and allophycocyanin (APC)-conjugated CD1d tetramers loaded with α-galactosylceramide (NIH Tetramer Facility, Emory University, Atlanta, GA) to analyze αβ T cell, γδT cell, NK cell, and NKT cell populations. To analyze neutrophil, B cell, and macrophage populations, samples were stained with CD19 FITC (MB19.1; eBioscience), Ly6G PE (1A8; BD Biosciences), CD11c PerCP Cy5.5 (N418; eBioscience), and F4/80 APC (BM8; eBioscience) or isotype IgG2a,k APC (R35-95; BD Biosciences). Cells were examined using a BD FACSCalibur flow cytometer and analyzed using CellQuest software (BD Biosciences).

### Processing of lungs and BALF for bacterial burden and cytokine analysis.

Serial dilutions of 10 μl of the first 1 ml of collected BALF were plated on LB agar plates and incubated for 24 h at 37°C. Colonies were counted to determine CFU. The remaining BALF was centrifuged at 470 × *g* for 5 min, and the supernatant was stored at −80°C for cytokine analysis. Lungs were isolated postinfection and homogenized in 50 mM HEPES buffer (Sigma-Aldrich) with 0.1 mg/ml soybean trypsin inhibitor for 20 s. Serial dilutions of 10 μl of lung homogenates were plated on LB agar plates for bacterial counting. Colonies were counted to determine CFU per milligram of tissue. Erythrocytes in the homogenates were lysed in lysis buffer. The homogenates were centrifuged at 18,000 × *g* for 30 min at 4°C, and the supernatant was stored at −80°C for cytokine analysis.

### Cytokine detection.

Cytokine levels in the supernatant of extracted lung tissue and BALF were measured using a mouse Th1/Th2 10plex FlowCytomix multiplex bead assay kit (eBioscience). Data were acquired using a CytoFlex flow cytometer (Beckman Coulter) and FCS Express Flow 6 software. MIP-2, KC, and IL-1β were measured by enzyme-linked immunosorbent assay (ELISA) using antibody pairs and reagents purchased from R&D Systems. Keratinocyte growth factor was measured using an ELISA kit from RayBiotech.

### Statistical analysis.

Unless otherwise noted, data are expressed as the mean ± the standard error of the mean. Statistical analysis was performed on pooled data using GraphPad Prism 8.1.2. Survival curves were compared by Mantel-Cox log-rank test. Bacterial CFU were compared by nonparametric Kruskal-Wallis analysis followed by Dunn’s posttest. Other data sets were compared by parametric analysis of variance with Tukey’s posttest. *P* values of <0.05 were considered significant.

## Supplementary Material

Supplemental file 1
