# Supplementary material for: Mice Lacking γδ T Cells Exhibit Impaired Clearance of Pseudomonas aeruginosa Lung Infection and Excessive Production of Inflammatory Cytokines
Source: Infect Immun. 2020 May 20;88(6):e00171-20. doi: 10.1128/IAI.00171-20 (PMC7240087; doi:10.1128/IAI.00171-20)
Supplement: Supplemental file 1 [file IAI.00171-20-s0001.pdf]

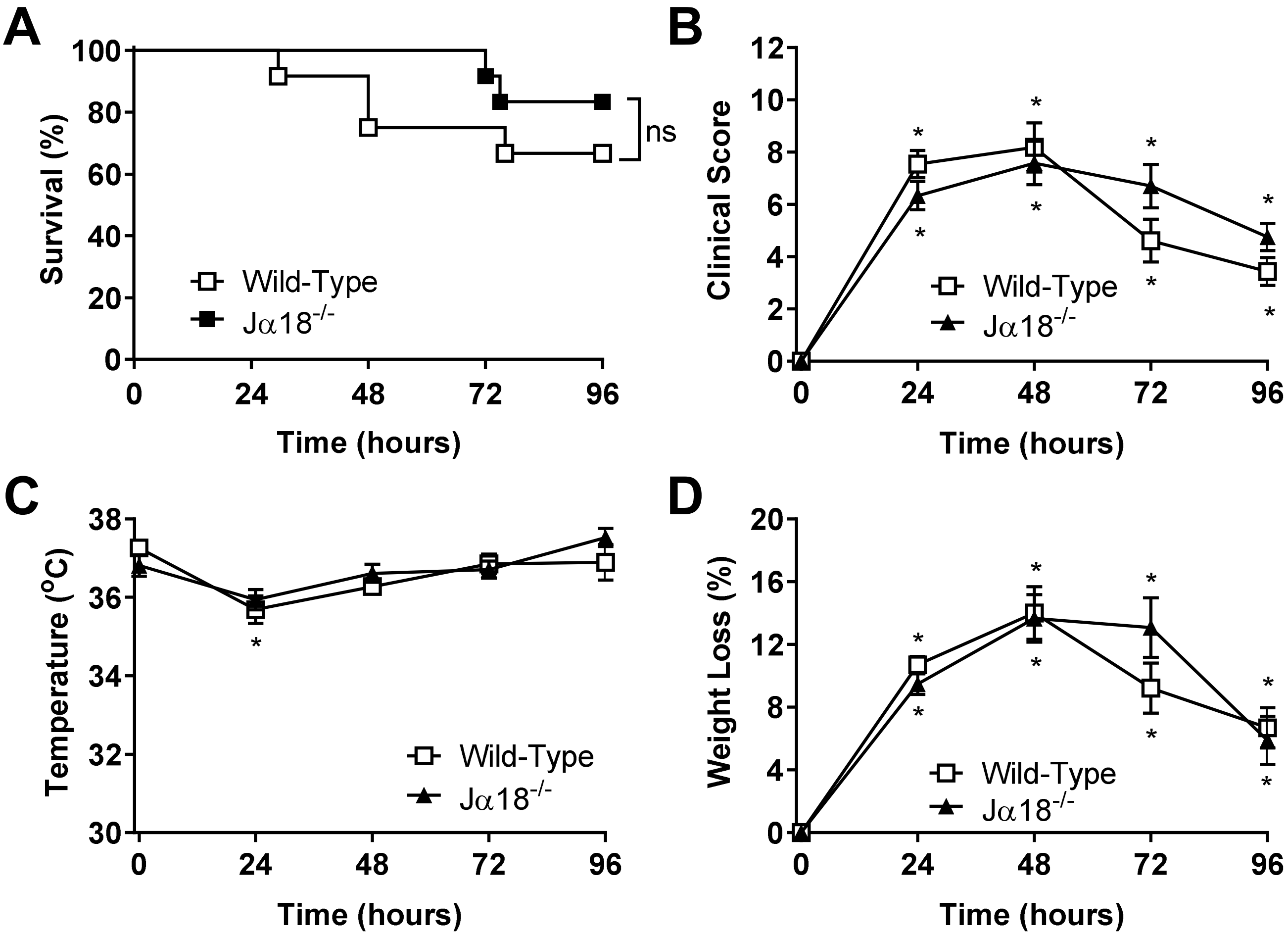

Figure S1. Survival and clinical parameters in wild-type and Jα18$^{-/-}$ mice infected with *P. aeruginosa*. (A) Survival curves, (B) clinical scores, (C) rectal temperature, and (D) weight loss were measured in wild-type and TCR$\delta^{-/-}$ mice intranasally inoculated with 1.8×10^7 CFU PAK (n=12 per group, pooled from 2 separate experiments). Survival curves were compared by Mantel-cox log-rank test (ns = not significant). Other parameters were assessed by Tukey's multiple-comparison test, * $P < 0.05$ compared with time 0; † $P < 0.05$ compared with wild-type mice.

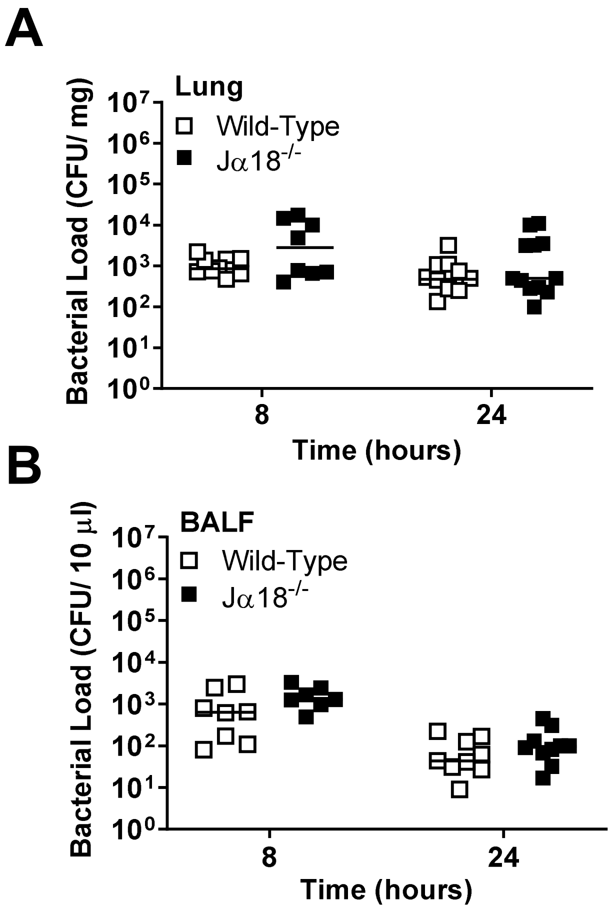

Figure S2. Bacterial load in wild-type and Jα18$^{-/-}$ mice infected with *P. aeruginosa.* Wild-type and Jα18$^{-/-}$ mice were infected intranasally with 1.8×10^7 CFU PAK. CFU were evaluated in (A) lung homogenates and (B) BALF at 8 or 24 hours after infection (n= 8-10 per group). Each symbol represents an individual animal, horizontal lines represent the median. Using Dunn's multiple-comparison test, * $P < 0.05$ compared with 0 hours; † $P < 0.05$ compared with wild-type mice.
